# Genomic analysis of *Skermanella stibiiresistens* type strain *SB22*^T^

**DOI:** 10.4056/sigs.5751047

**Published:** 2014-04-20

**Authors:** Wentao Zhu, Jing Huang, Mingshun Li, Xiangyang Li, Gejiao Wang

**Affiliations:** State Key Laboratory of Agricultural Microbiology, College of Life Sciences and Technology, Huazhong Agricultural University, Wuhan, China

**Keywords:** *Skermanella stibiiresistens*, genome sequence, metabolism, flagella, chemotaxis

## Abstract

Members of genus *Skermanella* were described as Gram-negative, motile, aerobic, rod-shaped, obligate-heterotrophic bacteria and unable to fix nitrogen. In this study, the genome sequence of *Skermanella stibiiresistens* SB22^T^ is reported. Phylogenetic analysis using core proteins confirmed the phylogenetic assignment based on 16S rRNA gene sequences. Strain SB22^T^ has all the proteins for complete glycolysis, tricarboxylic acid cycle and pentose phosphate pathway. The RuBisCO encoding genes *cbbL1S1* and nitrogenase delta subunit gene *anfG* are absent, consistent with its inability to fix carbon and nitrogen, respectively. In addition, the genome possesses a series of flagellar assembly and chemotaxis genes to ensure its motility.

## Introduction

The type species for the genus *Skermanella* is *Skermanella parooens* ACM 2042^T^, which was originally proposed as *Conglomeromonas largomobilis subsp. parooensis* by Skerman *et al*. in 1983 [1]. Later, it was transferred to the genus *Skermanella* (family *Rhodospirillaceae*) on the basis of phylogenetic evidence and phenotypic characteristics, especially the inability to fix nitrogen [2,3]. At present, this genus comprises four validly published species, *Skermanella parooensis* [3], *Skermanella aerolata* [4], *Skermanella xinjiangensis* [5] and *Skermanella stibiiresistens* [6], which were isolated from fresh water, air, sandy soil and a coal mine, respectively.

*Skermanella* was characterized as a Gram-negative, non-spore-forming bacterium with unicellular and multicellular phases of growth, an obligate chemo-organotroph and facultative anaerobe, unable to fix nitrogen, and with a high DNA G+C content. To the best of our knowledge, genome information for *Skermanella* members is still not available. In this study, we present the draft genome sequence of a *Skermanella* type strain *S. stibiiresistens* SB22^T^ and compare it with the members of the related genera *Azospirillum* and *Rhodospirillum*.

### Classification and features

**Figure 1:**
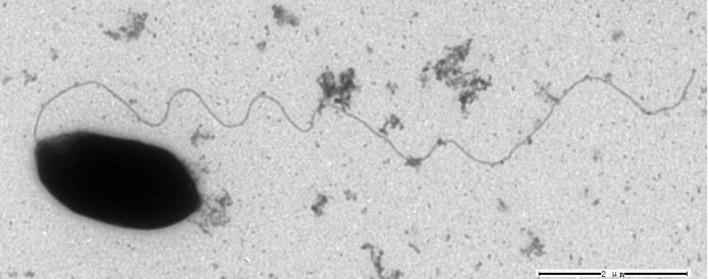
A transmission micrograph of *S. stibiiresistens* SB22^T^, made using a Hitachi H-7000FA transmission electron microscope operating at 100 kV. The scale bar represents 2 μm.

**Figure 2:**
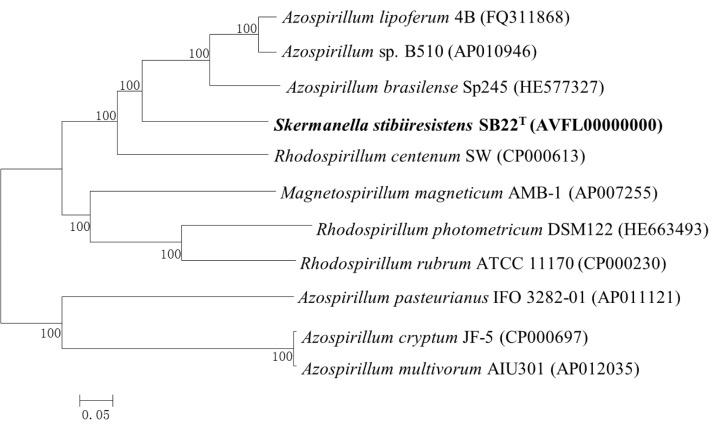
A NJ phylogenetic tree highlighting the position of *S. stibiiresistens* SB22^T^ with other completely sequenced strains that belong to the same family (*Rhodospirillaceae*). All protein FASTA files were obtained from NCBI. The corresponding GenBank accession numbers are displayed in parentheses. A total of 515 conserved proteins were identified using cluster algorithm tool MCL. The 515 amino acid sequences were aligned using Clustal W [15] and the NJ tree was built using MEGA 5.05 [16] with boot strap value of 1,000.

*S. stibiiresistens* SB22^T^ is Gram-negative, motile, rod-shaped with single polar flagella and non-sporulating (Figure 1). Ten related strains with complete genome sequences belong to *Azospirillum* (3 strains), *Rhodospirillum* (3 strains), *Acidiphilium* (2 strains), *Magnetospirillum* (1 strain) and *Acetobacter* (1 strain). A total of 515 conserved proteins were identified among them using cluster algorithm tool MCL (http://micans.org/mcl/) with default values and a neighbor joining (NJ) tree was built based on this set. The phylogenetic tree showed that strain SB22^T^ was closely related to the genera *Azospirillum* and *Rhodospirillum*, which was consistent with the taxonomy previously determined by 16S rRNA gene sequence analysis [6]. Classification and general features of *S. stibiiresistens* SB22^T^ are shown in Table 1. Figure 2 shows the phylogenetic neighborhood of *S. stibiiresistens* SB22^T^ in a core-protein based tree.

**Table 1:** Classification and general features of *S. stibiiresistens* SB22^T^ according to the MIGS recommendations [7].

**MIGS ID**	**Property**	**Term**	**Evidence code**	
	Current classification	Domain *Bacteria*		TAS [8]
		Phylum *Proteobacteria*		TAS [9]
		Class *Alphaproteobacteria*		TAS [10,11]
		Order *Rhodospirillales*		TAS [12,13]
		Family *Rhodospirillaceae*		TAS [12,13]
		Genus *Skermanella*		TAS [3,4,6]
		Species *Skermanella stibiiresistens*		TAS [6]
		Type strain SB22^T^		TAS [6]
	Gram stain	Negative		TAS [6]
	Cell shape	Rod-shaped		TAS [6]
	Motility	Motile		TAS [6]
	Sporulation	Non-sporulating		TAS [6]
	Optimum temperature	4-37ºC		TAS [6]
	Carbon source	D-glucose, D-ribose, rhamnose, L-proline, salicin, inositol, DL-lactate, L-alanine, malic acid, potassium 2-ketogluconate and 3-hydroxybutyric acid		TAS [6]
	Energy source	Chemoorganotroph		TAS [6]
	Terminal electron receptor	Molecular oxygen		TAS [6]
MIGS-6.2	pH	5-9		TAS [6]
MIGS-22	Oxygen	Aerobic		TAS [6]
MIGS-15	Biotic relationship	Free-living		NAS
MIGS-14	Pathogenicity	Non-pathogenic		NAS
MIGS-4	Geographic location	Jixi coal mine of Jixi City, Heilongjiang Province, northeast China		TAS [6]
MIGS-5	Sample collection time	2011		TAS [6]
MIGS-4.1 MIGS-4.2	Latitude Longitude	N45°18' E130°57'		TAS [6] TAS [6]
MIGS-4.3	Depth	Surface sandy soil		TAS [6]
MIGS-4.4	Altitude	Not reported		

Evidence codes - IDA: Inferred from Direct Assay; TAS: Traceable Author Statement; NAS: Non-traceable Author Statement. These evidence codes are from the Gene Ontology project [14]. If the evidence is IDA, then the property was directly observed for a live isolate by one of the authors or an expert mentioned in the acknowledgements.

## Genome sequencing information

### Genome project history

*S. stibiiresistens* SB22^T^ was sequenced by Majorbio Bio-pharm Technology Co., Ltd, Shanghai, China. The draft genome sequence was deposited in NCBI with contigs less than 200 bp cut off. The GenBank accession number is AVFL00000000. A summary of the genome sequencing project information is shown in Table 2.

**Table 2:** Genome sequencing project information of *S. stibiiresistens* SB22^T^

**MIGS ID**	**Property**	**Term**
MIGS-31	Finishing quality	High-quality draft
MIGS-28	Libraries used	Illumina Paired-End library (300 bp insert size)
MIGS-29	Sequencing platform	Illumina Hiseq2000
MIGS-31.2	Sequencing coverage	184.5 ×
MIGS-30	Assemblers	SOAPdenovo v1.05
MIGS-32	Gene calling method	GeneMarkS^+^
	GenBank date of release	February 23, 2014
	NCBI project ID	AVFL00000000
MIGS-13	Source material identifier	SB22^T^
	Project relevance	Genome comparison

### Growth condition and DNA isolation

*S. stibiiresistens* SB22^T^ was grown aerobically in R2A medium at 28°C for 2 days. It can also grow on LB medium under the same conditions. The strain was apricot-colored after incubated 72 h at 28°C on R2A agar. The DNA was isolated using the QiAamp kit according to the manufacturer’s instruction (Qiagen, German).

### Genome sequencing and assembly

The Illumina Hiseq2000 technology with Paired-End (PE) library strategy was used to determine the sequence of *S. stibiiresistens* SB22^T^. A total of 7,588,874 x 2 high quality reads totaling 1,454,191,294 bp data with an average coverage 184.5 x was generated. Illumina sequencing data was assembled with SOAPdenovo, version 1.05 (http://soap.genomics.org.cn/). The initial draft assembly contained 7,879,677 bp in 257 contigs. Then the draft genome sequence was deposited to the NCBI with contigs less than 200 bp nucleotides cut off.

### Genome annotation

The draft genome sequence was deposited to NCBI and was annotated though the Prokaryotic Genome Annotation Pipeline (PGAP), using the Best-placed reference protein set and the gene caller GeneMarkS^+^. Signal peptides and transmembrane helices were predicted by SignalP [17] and SOSUI [18], respectively. The WebMGA-server [19] was used to identify the Clusters of Orthologs Groups (COG).

## Genome properties

**Figure 3:**
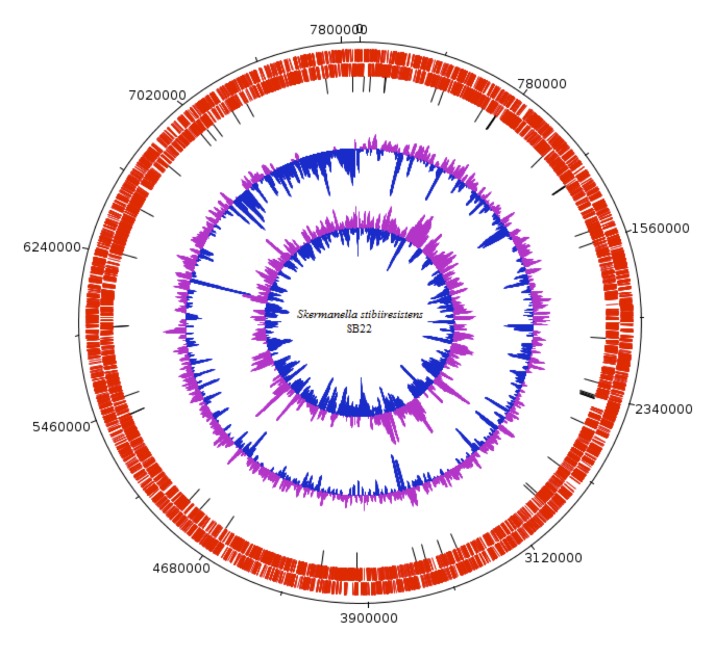
A graphical circular map of *S. stibiiresistens* SB22^T^. From outside to the center: Genes on forward strand, Genes on reverse strand, tRNA genes, GC content and GC skew.

The final whole genome of *S. stibiiresistens* SB22^T^ was 7,868,338 bp long in 190 contigs (with PEGs) with an average GC content of 65.88%. Of the total 7,378 predicted genes, 7,269 were protein-coding genes and 63 were RNA genes. A total of 5,176 protein-coding genes (71%) were assigned with putative functions with the remaining was annotated as hypothetical proteins. The property and the statistics of this genome are summarized in Table 3. We reordered the contigs using MAUVE, version 2.3 [20] with the complete genome sequence of *Rhodospirillum centenum* …( ) as a reference and a graphical circular map of *S. stibiiresistens* SB22^T^ is shown in Figure 3. The distribution of genes into COGs functional categories is shown in Table 4.

**Table 3:** Nucleotide content and gene count level in genome of *S. stibiiresistens* SB22^T^

**Attribute**	**Value**	**% of Total^a^**
Genome size (bp)	7,868,388 bp	100
Number of contigs	190	
Contig N50	214,710 bp	
Total genes	7,378	100
Protein-coding genes	7,269	98.52
Pseudo genes	46	0.62
RNA genes	63	0.86
Frame shifted genes	13	
DNA coding region (bp)	6,849,861	87.06
Protein-coding genes with function prediction	5,176	71.21
Protein-coding genes assigned to COGs	5,877	80.85
Protein-coding genes with transmembrane helices	1,673	23.02
Protein-coding genes with signal peptides	523	7.19
CRISPR repeats	2	

^a^ The total is based on either the size of the genome in base pairs or the total number of protein coding genes in the annotated genome.

**Table 4:** Number of protein-coding genes associated with the 25 general COG functional categories in *S. stibiiresistens* SB22^T^ genome.

**Code**	**Value**	**%age^a^**	**COG Category**
J	188	2.59	Translation, ribosomal structure and biogenesis
A	1	0.01	RNA processing and modification
K	478	6.58	Transcription
L	240	3.30	Replication, recombination and repair
B	6	0.08	Chromatin structure and dynamics
D	50	0.69	Cell cycle control, cell division, chromosome partitioning
Y	0	0.00	Nuclear structure
V	73	1.00	Defense mechanisms
T	651	8.96	Signal transduction mechanisms
M	389	5.35	Cell wall/membrane/envelope biogenesis
N	147	2.02	Cell motility
Z	0	0.00	Cytoskeleton
W	0	0.00	Extracellular structures
U	76	1.05	Intracellular trafficking, secretion, and vesicular transport
O	195	2.68	Posttranslational modification, protein turnover, chaperones
C	405	5.57	Energy production and conversion
G	508	6.99	Carbohydrate transport and metabolism
E	678	9.33	Amino acid transport and metabolism
F	101	1.39	Nucleotide transport and metabolism
H	230	3.16	Coenzyme transport and metabolism
I	267	3.67	Lipid transport and metabolism
P	383	5.27	Inorganic ion transport and metabolism
Q	226	3.11	Secondary metabolites biosynthesis, transport and catabolism
R	787	10.83	General function prediction only
S	573	7.88	Function unknown
-	617	8.49	Not in COGS

^a^ The total is based on the total number of protein coding genes in the annotated genome.

## Profiles of metabolic networks and pathways

**Figure 4:**
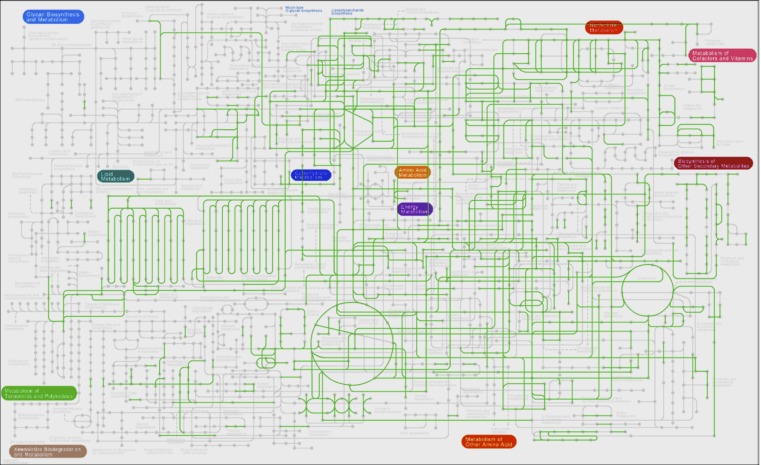
Metabolic pathways of *S. stibiiresistens* SB22^T^ as predicted using KEGG. Green lines indicate pathways that are possessed by this strain.

The Kyoto Encyclopedia of Genes and Genomes (KEGG) [21] was used to reconstruct the pathways of *S. stibiiresistens* SB22^T^ [Figure 4]. The metabolic pathways suggest that strain SB22^T^ possess necessary encoding genes for central carbohydrate metabolism, such as glycolysis, the TCA cycle and the pentose phosphate pathway, to support basic growth. But it has relatively few lipid metabolism pathways. Only one integrated pathway that could synthesize tetracosanoyl-CoA from acyl-CoA is found in cytoplasm. This result indicates that strain SB22^T^ might have certain limitations in some lipid utilization.

### Genes involved in carbon fixation

Genus *Skermanella* belongs to family *Rhodospirillaceae*, but *Skermanella* species cannot fix carbon as *Rhodospirillum centenum* [22] and *Azospirillum amazonense* [23] do. Genomic analysis of *S. stibiiresistens* SB22^T^, shows that the RuBisCO encoding genes *cbbL1S1* [24,25] are not present, which is in agreement with the strain’s inability to fix carbon.

### Genes involved in nitrogen metabolism

Strain *S. stibiiresistens* SB22^T^ is closely related to some species of genera *Azospirillum* and *Rhodospirillum* [6]. Genus *Rhodospirillum* is described as a photosynthetic non-sulfur purple bacterium that favors growth in an anoxygenic, photosynthetic, nitrogen-fixing environment [26]. Some aerobic nitrogen fixing strains of *Azospirillum* have significant effects on crop plants [27]. But genus *Skermanella* is unable to fix nitrogen under microaerophilic conditions [3,4,6]. Even though nitrogenase genes *nifDKH* are present in the genome of *S. stibiiresistens* SB22^T^, we found that the nitrogenase delta subunit gene *anfG* is absent.

### Flagella and chemotaxis

**Figure 5:**
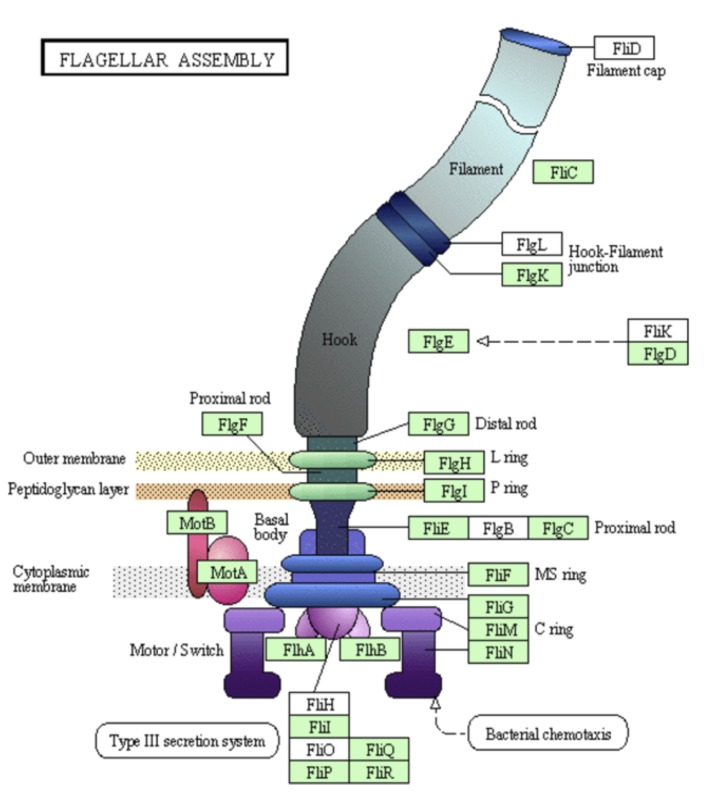
The KEGG flagellar assembly map. Green labels represent the flagella proteins that are encoded on the *S. stibiiresistens* SB22^T^ genome.

Many flagella genes were identified in *S. stibiiresistens* SB22^T^ genome. A KEGG map demonstrates that this strain possesses a series of genes belonging to the families *flg*, *fli* and *flh* in flagellar assembly (Figure 5). These genes enable strain SB22^T^ to move to a more suitable environment. In the same region of the genome as the flagella genes, we also identified genes encoding the central signal transduction pathway for chemotaxis (*che*), such as the conserved *cheAWYBR* genes and a group of transmembrane chemoreceptors (known as MCPs or methyl-accepting proteins), which are present in nearly all motile bacteria [23]. The MCPs may sense environment signals and transfer them to the signal transduction histidine kinase CheA, whose activity is positively regulated by CheW. CheA in turn phosphorylates a response regulator CheYVB, which controls the rotational direction of the flagella motor [28].
